# Case Report: Management of a *BCR-ABL1*-positive high-risk rhabdomyosarcoma patient using tyrosine kinase inhibitors

**DOI:** 10.3389/fmed.2025.1693688

**Published:** 2025-10-13

**Authors:** Fan Li, Dapeng Jiang, Yu Liu, Minzhi Yin, Yumin Zhong, Min Xu, Anan Zhang, Yali Han

**Affiliations:** ^1^Department of Oncology, Shanghai Children's Medical Center, Shanghai Jiao Tong University School of Medicine, Shanghai, China; ^2^Institute of Translational Medicine, Shanghai Children's Medical Center, Shanghai Jiao Tong University School of Medicine, Shanghai, China; ^3^Department of Pathology, Shanghai Children's Medical Center, Shanghai Jiao Tong University School of Medicine, Shanghai, China; ^4^Department of Radiology, Shanghai Children's Medical Center, Shanghai Jiao Tong University School of Medicine, Shanghai, China

**Keywords:** rhabdomyosarcoma, *BCR-ABL1*, tyrosine kinase inhibitor, chemotherapy, case report

## Abstract

Rhabdomyosarcoma represents a prevalent type of soft tissue sarcoma encountered in pediatric patients. Despite multimodal intensified therapies encompassing surgical intervention, chemotherapy, and radiotherapy, the prognosis for patients with high-risk rhabdomyosarcoma remains notably unfavorable. To date, no definitive and efficacious molecularly targeted therapies have been established. This report describes the first documented case of a rhabdomyosarcoma patient harboring a positive *BCR-ABL1* fusion gene. At the time of initial diagnosis, the patient presented with a primary tumor in the right thigh and extensive metastatic involvement affecting both lungs, pleura, mediastinum, pelvic cavity, and the right inguinal region, resulting in the classification of the case as high-risk. In addition to conventional multimodal therapy, early intervention using tyrosine kinase inhibitors was implemented, leading to the achievement of an early complete response.

## 1 Introduction

Rhabdomyosarcoma represents a frequently encountered soft tissue sarcoma in children. Previous research indicates that despite undergoing intensified treatment modalities encompassing surgery, chemotherapy and radiotherapy, high-risk patients with two or more risk factors had a 3-year event-free survival of only 20%, signifying a very poor outcome (1). In recent years, molecular targeted therapy has progressively emerged as a novel therapeutic exploration for rescuing children with advanced rhabdomyosarcoma. *PAX3-FOXO1* and *PAX7-FOXO1* are significant driver genes and prognostic indicators for alveolar rhabdomyosarcoma (2). The pathogenesis of other pathological subtypes might be associated with somatic mutations within the RAS signaling pathway and 11p15.5 heterozygous deletion (3, 4). Nevertheless, no explicit and effective molecular targeted drugs have been identified thus far. Prior studies have demonstrated that mTOR inhibitor temsirolimus can ameliorate short-term prognosis (5); however, the latest research findings reveal that addition of temsirolimus to chemotherapy did not improve event-free survival in patients with intermediate-risk rhabdomyosarcoma (6). Hence, for children with intermediate and high-risk rhabdomyosarcoma, identifying specific genetic alterations might be the crux for the success of molecular targeted therapy. This paper reports a case of a pediatric patient with high-risk rhabdomyosarcoma harboring a positive *BCR-ABL1* fusion gene, which represents the first such discovery to date. At the initial diagnosis, this patient presented with widespread disseminated metastasis. After undergoing combined therapy involving chemotherapy, surgery, radiotherapy, and tyrosine kinase inhibitors (TKIs), an early complete response (CR) was attained.

## 2 Case presentation

**Figure 1:**
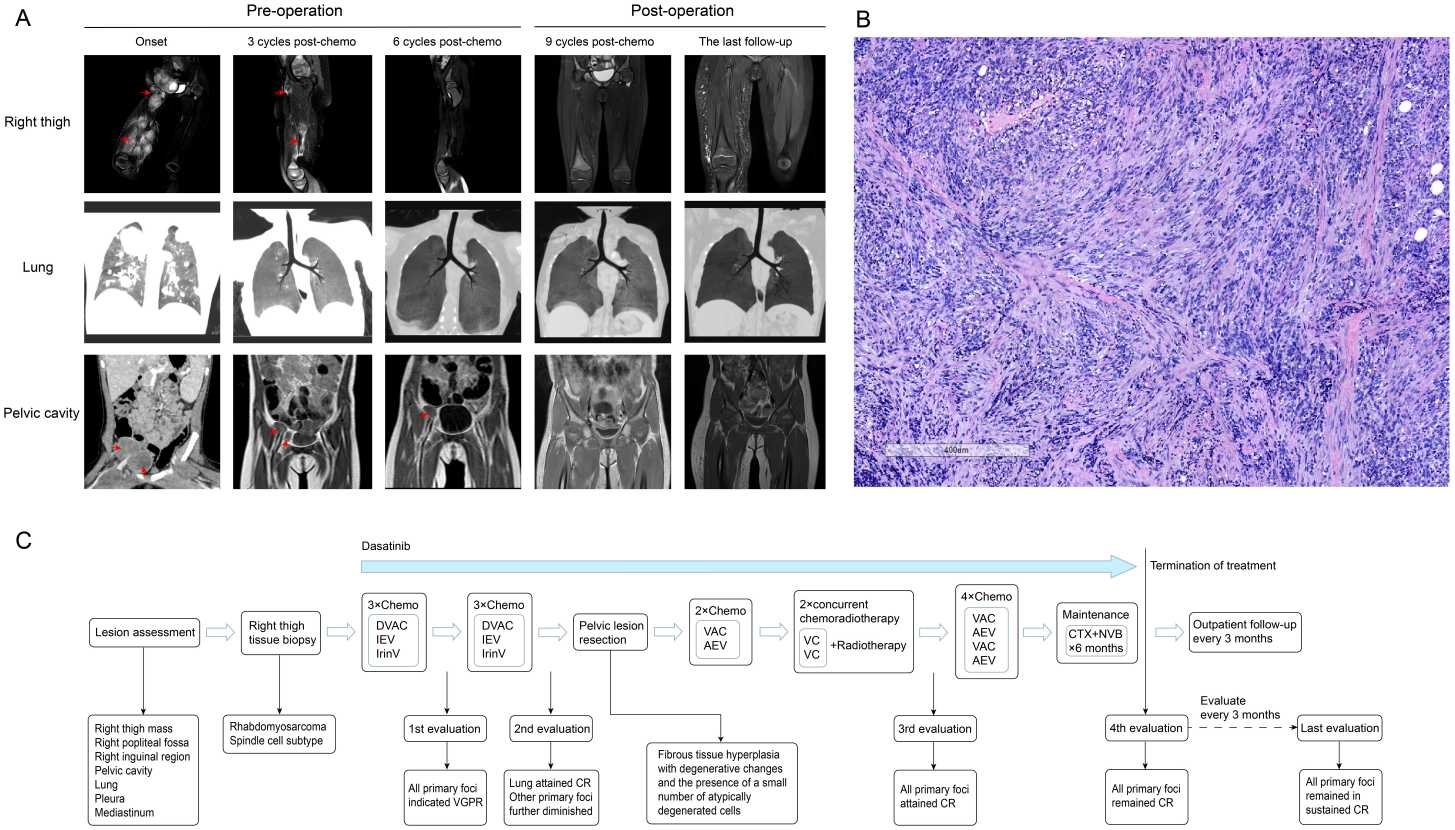
Imaging findings, staining of pathological sections, and the flowchart of diagnosis, therapy, and follow-up for the patient. **(A)** (1) Initial imaging assessment: enhanced MRI (T2-weighted) of the right thigh revealed extensive mass-like abnormal signals within the subcutaneous soft tissue of the right thigh (predominantly in the muscle layer), the right popliteal fossa, the right inguinal region, and the right pelvic cavity. Marked heterogeneous enhancement was observed after contrast administration. Chest CT demonstrated multiple nodular opacities in both lungs, the pleura, and the mediastinum with clear boundaries. The enhanced CT of the pelvic cavity revealed multiple mass-like shadows in the right pelvic cavity and the inguinal region, some of which were fused. The pelvic mass was indistinctly demarcated from the adjacent iliopsoas muscle. Heterogeneous enhancement was noted after contrast administration. (2) Evaluation after three courses of chemotherapy: chest CT, pelvic and right thigh MRI (T2-weighted) scans demonstrated a substantial reduction in all primary lesions, indicating a very good partial response (VGPR) status. (3) Evaluation after six courses of chemotherapy: chest CT indicated a complete response (CR) in lung lesions, while MRI (T2-weighted) of the pelvis and right thigh demonstrated further reduction in the primary lesion. (4) Initial evaluation following surgical and radiotherapy treatment: all primary lesions demonstrated a CR. (5) Final follow-up evaluation: all primary lesions remained in sustained CR. **(B)** This panel presents the hematoxylin and eosin (H&E) stained histopathological image of the biopsy specimen obtained from the lesion on the patient's right thigh. Immunohistochemistry results were as follows: Desmin positive, Vimentin positive, MyoD1 positive, INI-1 positive, Myogenin partially positive, S100 positive, SOX-10 negative, CD99 negative, SMA positive, ALK negative, MSA positive, KI-67 positive, and CD34 negative. The *VGLL2-CITED2* fusion was identified using next-generation sequencing. **(C)** In accordance with the Shanghai Children's Medical Center's standard treatment protocol for rhabdomyosarcoma (SCMC-RS-2018), the patient was diagnosed with rhabdomyosarcoma via pathological biopsy, with the primary tumor located in the soft tissue of the right thigh. Imaging studies confirmed multiple distant metastases in both lungs, pleura, mediastinum, pelvic cavity, and the right inguinal region, leading to the classification of the patient as high-risk for therapeutic management. The figure illustrates the comprehensive treatment process, lesion evaluation, and post-discontinuation follow-up assessment for this patient.

The patient was a 2-year and 6-month-old girl who presented to our hospital with progressive swelling and pain in the right lower extremity. A mass was detected on the inner side of the right leg, which gradually enlarged, accompanied by tenderness in the right lower extremity that intensified during movement. Physical examination revealed significant swelling in the right buttock, thigh, and calf, with elevated skin temperature. Multiple round-shaped masses were palpable in the right thigh, firm in texture, with indistinct boundaries. The right hip and right knee had restricted movement due to pain. Imaging studies demonstrated the presence of a large mass in the subcutaneous soft tissues of the right thigh, with multiple metastases in both lungs, pleura, mediastinum, right popliteal fossa, pelvic cavity, and the right inguinal region (Figure 1A). The bone marrow cytology and immunophenotyping results were both negative. Subsequently, the patient underwent a biopsy of the right thigh mass. Postoperative pathological examination, incorporating morphological and immunohistochemical analyses, as well as confirmation of positive *VGLL2-CITED2* fusion, established the diagnosis of spindle cell/sclerosing rhabdomyosarcoma (Figure 1B). The pathological diagnosis was independently confirmed by two experienced pathologists, each with over 10 years of expertise in pediatric tumor pathology, yielding concordant results. Comprehensive clinical and pathological evaluations were performed, including differential diagnoses that effectively ruled out other potential disease entities such as other subtypes of rhabdomyosarcoma, rhabdoid tumor, Ewing sarcoma/primitive neuroectodermal tumor, spindle cell tumors, and histiocytic sarcoma. In accordance with the Shanghai Children's Medical Center's treatment protocol for rhabdomyosarcoma (SCMC-RS-2018), the patient was categorized into the high-risk group for therapeutic management.

**Figure 2:**
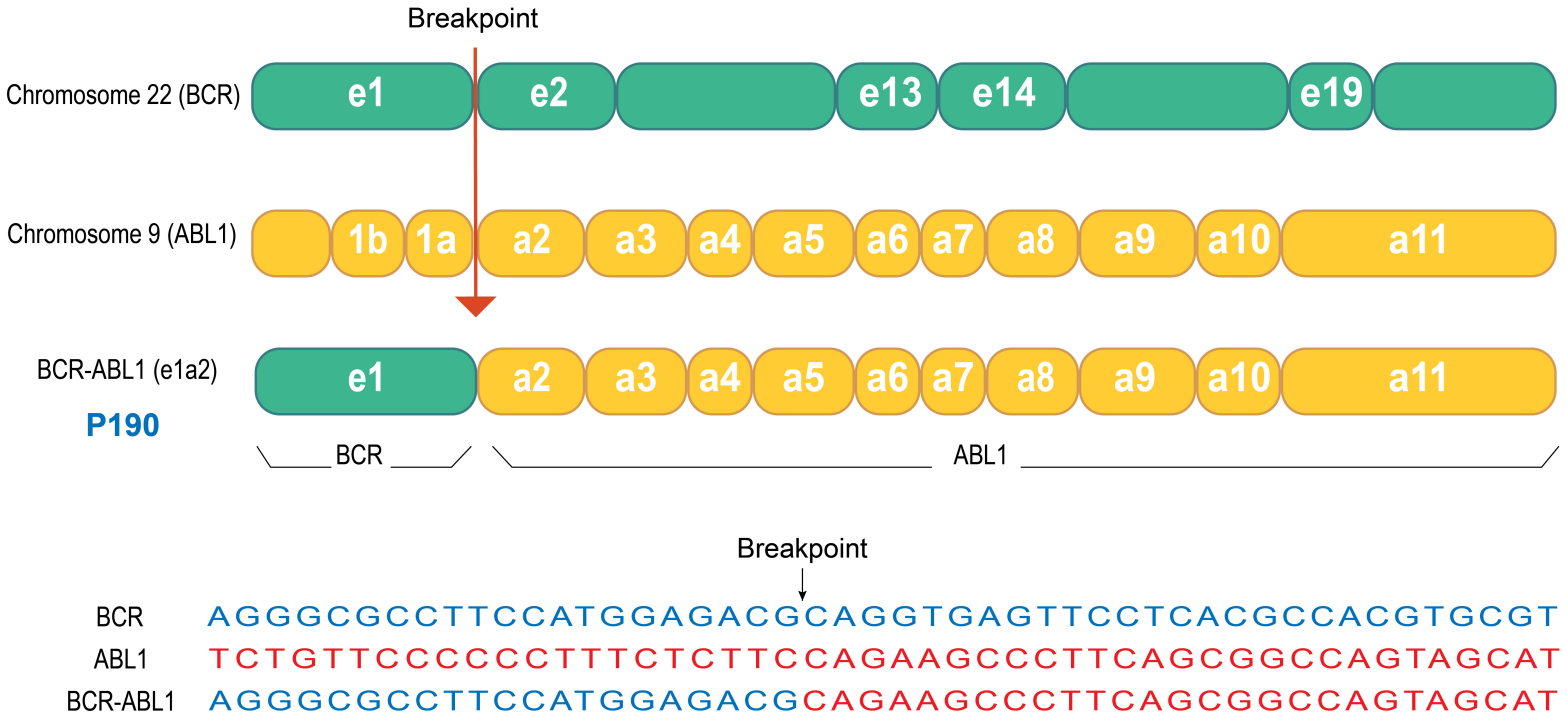
Schematic representation of the *BCR-ABL1* gene fusion in this patient. Next-generation sequencing analysis of the tumor tissue from this patient revealed the presence of the *BCR-ABL1* fusion gene, specifically the p190 isoform. As illustrated in the figure, the fusion event involved exon 1 (e1) of the *BCR* gene on chromosome 22 and exon 2 (a2) of the *ABL1* gene on chromosome 9, resulting in the e1a2 fusion variant.

Chemotherapy was initiated after biopsy, and the regimen and courses are shown in Table 1 and Figure 1C. The results of next-generation sequencing performed on the tumor tissue DNA confirmed the presence of a *BCR-ABL1* (p190) fusion (Figure 2). Dasatinib was initiated at an initial dose of 60 mg/m^2^ once daily for oral administration. Following the completion of three cycles of chemotherapy, a comprehensive imaging assessment indicated a very good partial response (VGPR). By the conclusion of the sixth cycle, lung lesions were re-evaluated and found to have achieved CR, with remaining lesions demonstrating further reduction. A surgical resection of the residual pelvic mass was subsequently conducted. Postoperative pathological examination revealed fibrous tissue hyperplasia with degenerative changes, the presence of a small number of atypically degenerated cells, and inflammatory cell infiltration within the stroma. Postoperatively, chemotherapy concurrently with radiotherapy to the right thigh and the right pelvic wall was initiated, with a total radiation dose of 50.4 Gy. Following the completion of radiotherapy, a re-evaluation confirmed that all primary lesions had achieved CR. Maintenance therapy was initiated with a combination of cyclophosphamide and vinorelbine, administered over a 6-month treatment period. During this treatment period, two comprehensive assessments were performed, both confirming CR. The treatment of TKI was discontinued after maintenance therapy. Following cessation of treatment, the patient underwent regular outpatient follow-up every 3 months. As of the most recent evaluation conducted 1 year after discontinuation of the drug, the patient remains in sustained CR.

**Table 1:** Chemotherapy regimen: drugs, dosage, and days.

**Regimen**	**Drugs**	**Dosage**	**Days**
DVAC	Doxorubicin	35 mg/m^2^	1 and 8
	Cyclophosphamide	400 mg/m^2^	1–3
	Vincristine	1.5 mg/m^2^	0 and 7
	Dactinomycin	0.045 mg/kg	1
IEV	Ifosfamide	1.8 g/m^2^	1–5
	Etoposide	100 mg/m^2^	1–5
	Vincristine	1.5 mg/m^2^	0 and 7
IrinV	Irinotecan	50 mg/m^2^	1–5
	Vincristine	1.5 mg/m^2^	0 and 7
VAC	Cyclophosphamide	1.2 g/m^2^	1
	Vincristine	1.5 mg/m^2^	0 and 7
	Dactinomycin	0.045 mg/kg	1
AEV	Dactinomycin	0.045 mg/kg	1
	Etoposide	100 mg/m^2^	1–5
	Vincristine	1.5 mg/m^2^	0 and 7
VC	Vincristine	1.5 mg/m^2^	0 and 7
	Cyclophosphamide	1.2 g/m^2^	1
Maintenance (six 4-week cycles)	Vinorelbine	25 mg/m^2^	1, 8, and 15
	Cyclophosphamide	25 mg/m^2^	1–28

The patient's legal guardian consented to the disclosure of the patient's biological and imaging data, and signed the informed consent.

## 3 Discussion

With the discovery of the Philadelphia chromosome in 1960 and the identification of the ABL proto-oncogene in the 1980s, the role of ABL protein family kinases in tumorigenesis was revealed (7, 8). Among fusion gene families, *BCR-ABL1* stands as the first cancer-related fusion gene to be identified. The encoded *BCR-ABL1* fusion protein exhibits constitutive tyrosine kinase activity, which activates multiple downstream signaling pathways, resulting in uncontrolled cell proliferation, aberrant differentiation, and inhibition of apoptosis, thereby driving the development of chronic myeloid leukemia (CML). Since the tyrosine kinase fusion protein serves as a pivotal driver of malignant cell proliferation in CML, the presence of the Philadelphia chromosome or *BCR-ABL1* fusion remains the gold standard for diagnosing CML (9). In addition to CML, the *BCR-ABL1* fusion has also been identified in a subset of acute lymphoblastic leukemia (ALL) and acute myeloid leukemia (AML) cases, as well as in rare instances of other hematological malignancies, including lymphoma, histiocytic sarcoma, and myeloid sarcoma, suggesting potential clonal relationships among different types of hematological tumors (10–14). The *BCR-ABL1* fusion is exceptionally rare in solid tumors of non-hematopoietic origin. A previous study identified *BCR-ABL1* fusion in a patient with glioblastoma, but treatment with the TKI imatinib did not alter the patient's ultimate survival outcome (15).

TKIs act via competitive inhibition at the ATP-binding site of the *BCR-ABL1* oncoprotein, which results in the inhibition of phosphorylation of proteins involved in cell signal transduction (9), thereby inducing apoptosis in tumor cells. Nevertheless, it is observed in clinical practice that the therapeutic response to TKIs in *BCR-ABL1* positive ALL and AML is less sensitive compared with that in CML (16, 17). Consequently, the primary treatment modalities for these conditions still largely depend on chemotherapy and hematopoietic stem cell transplantation. Till now, it has generally been posited that, aside from CML and *BCR-ABL1* positive ALL, *BCR-ABL1* does not function as a pivotal driver gene influencing tumor cell proliferation, differentiation, and inhibition of apoptosis in other hematological malignancies, and may represent a rare subtype or an incidental genetic aberration in non-hematopoietic solid tumors.

To date, there have been no documented reports of *BCR-ABL1* fusion in soft tissue sarcomas, and its association with these tumors, including rhabdomyosarcoma, remains to be elucidated. In this case, the diagnosis of spindle cell/sclerosing rhabdomyosarcoma was confirmed based on the histopathological characteristics, immunohistochemical analysis, and positive *VGLL2-CITED2* molecular detection. Imaging findings revealed extensive metastases, which led to the patient being classified as high-risk. It is important to highlight that in prior studies, the *VGLL2-CITED2* fusion typically presents with spindle cell morphology and almost never results in distant metastasis (18, 19). However, in this instance, the patient exhibited widespread metastatic disease, and demonstrated high invasiveness. A notable discrepancy was observed between the clinical manifestations and the pathological molecular characteristics.

Through gene sequencing of the tumor tissue, an unexpected *BCR-ABL1* (p190) fusion gene was identified. P190 is one of the subtypes of *BCR-ABL1*, arising from the fusion of *BCR* exon 1 (e1) and *ABL1* exon 2 (a2), designated as e1a2. This isoform is identified in two-thirds of Philadelphia chromosome-positive ALL cases, but it is rarely observed in CML (16). We hypothesize that the highly aggressive nature of this tumor may be attributed to the oncogenic effects of *BCR-ABL1*. To further validate our hypothesis and investigate potential therapeutic benefits, we incorporated the second-generation TKI dasatinib into this patient's treatment regimen. Surprisingly, this patient exhibited a very good early response to the treatment. The primary lesion showed a VGPR after the initial three courses of chemotherapy. By the sixth course, the pulmonary lesions had achieved CR. After completion of radiotherapy, all lesions had reached CR. While it is currently not feasible to conclusively determine from a single case whether *BCR-ABL1* directly drives the proliferation and migration of tumor cells as it does in CML, the significant enhancement of treatment response by TKIs through *BCR-ABL1* inhibition suggests that, in this exceptionally rare case, *BCR-ABL1* may play a pivotal role in the highly aggressive pathogenesis of rhabdomyosarcoma. To further validate this hypothesis, additional case data must be accumulated and more in-depth research should be conducted.

We have observed the concurrent presence of the *BCR-ABL1* and *VGLL2-CITED2* fusions in this case. The *VGLL2-CITED2* fusion is an intrachromosomal rearrangement occurring on chromosome 6 and is recognized as a well-defined molecular subtype (19). In contrast, the *BCR-ABL1* fusion arises from an interchromosomal translocation between chromosomes 9 and 22 (9) and is not linked to chromosome 6. Given that these two fusion events involve distinct chromosomal loci, their co-occurrence in a single patient likely reflects independent and sporadic molecular events rather than a causal or mechanistic association. Nevertheless, comprehensive large-scale molecular profiling and the accumulation of additional clinical cases are necessary to fully evaluate any potential relationship between these genetic alterations.

Given that *BCR-ABL1* has not been previously documented in literature pertaining to rhabdomyosarcoma, it is typically not included as a standard diagnostic test for newly diagnosed rhabdomyosarcoma cases. However, the identification in this instance suggests that the prevalence of *BCR-ABL1* fusion positivity in rhabdomyosarcoma may have been underestimated. Conducting large-scale screening for the *BCR-ABL1* fusion gene could potentially uncover more such rare subtypes, thereby facilitating a deeper understanding of their mechanisms and providing robust, evidence-based support for precision targeted therapeutic approaches. Furthermore, we maintain that for patients diagnosed with high-risk, refractory, recurrent, and drug-resistant malignant solid tumors, it is essential to conduct comprehensive genomic analysis. This approach can facilitate the identification of unforeseen therapeutic targets and potentially offer additional treatment options for patients with advanced diseases.

## Data Availability

The original contributions presented in the study are included in the article/supplementary material, further inquiries can be directed to the corresponding author.
